# Flexible battery-less wireless glucose monitoring system

**DOI:** 10.1038/s41598-022-16714-1

**Published:** 2022-07-19

**Authors:** Saikat Banerjee, Gymama Slaughter

**Affiliations:** Center for Bioelectronics, Department of Electrical and Computer Engineering, Old Dominion University, Norfolk, VA 23528 USA

**Keywords:** Nanoscience and technology, Biomedical engineering, Electrical and electronic engineering

## Abstract

In this work, a low power microcontroller-based near field communication (NFC) interfaced with a flexible abiotic glucose hybrid fuel cell is designed to function as a battery-less glucose sensor. The abiotic glucose fuel cell is fabricated by depositing colloidal platinum (co–Pt) on the anodic region and silver oxide nanoparticles-multiwalled carbon nanotubes (Ag_2_O-MWCNTs) composite on the cathodic region. The electrochemical behavior is characterized using cyclic voltammetry and chronoamperometry. This glucose hybrid fuel cell generated an open circuit voltage of 0.46 V, short circuit current density of 0.444 mA/cm^2^, and maximum power density of 0.062 mW/cm^2^ at 0.26 V in the presence of 7 mM physiologic glucose. Upon device integration of the abiotic glucose hybrid fuel cell with the NFC module, the data from the glucose monitoring system is successfully transmitted to an android application for visualization at the user interface. The cell voltage correlated (r^2^ = 0.989) with glucose concentration (up to 19 mM) with a sensitivity of 13.9 mV/mM•cm^2^.

## Introduction

Continuous glucose monitoring is the most effective strategy to reduce the complications that might arise from elevated glucose levels in the body. Individuals with diabetes must frequently check glucose levels using either a finger prick test and/ or continuous glucose monitors (CGMs). An ideal sensor for monitoring glucose in the body must exhibit long-term stability and wirelessly communicate transitory changes in glucose levels with the patient or caregiver. Electrochemical transducers have garnered a lot of attention over the last few decades in the development of glucose-based biosensors^1–3^. Electrochemical transducers convert chemical or biological information, such as analyte concentration and overall composition, into a useful electrical signal. In addition, they have a wide range of benefits over other techniques, such as simple to construct and exhibit a quick reaction time with great limit of detection, selectivity, and sensitivity^4–6^.

The sensitivity of electrochemical biosensors is significantly enhanced by the conductive materials used in the design of electroactive area and nanomaterials has been extensively explored as sensing materials to boost the sensitivity and linear range of electrochemical biosensor^7^. Most electrochemical biosensors are designed to detect a wide range of analytes and are generally composed of an electrode sensing material modified with a biorecognition element or bioreceptor, such as enzymes, antibodies, or aptamers^8, 9^. Multiwalled and single-walled carbon nanotubes^6, 9^, semiconductor metal oxides^7, 10^, conducting polymers^11–13^, and graphene^14^ are some of the most used sensing materials. The application of nanoparticles or nanostructures, such as platinum, gold, and silver, continues to garner significant attention due to their exceptional electrochemical properties^15, 16^ to enhance direct and fast electron transfer from the bioreceptor to the current collector, as well as biosensor efficiency in the absence of mediators^17, 18^. These materials exhibit high volume to surface ratio and great biocompatibility and thus are appealing alternative to bioreceptors for biosensor development for wearable health monitoring devices^1, 19–21^.

Wearable healthcare devices have mostly focused on device miniaturization and wireless operation (e.g., Bluetooth and near-field communication (NFC))^22, 23^. Although the wearable device has mostly used Bluetooth technology, its large size and weight may affect wearability^22^. Ali et al. reported on the development of an implanted glucose monitoring device using Bluetooth low energy (BLE)^24^. Glucose data from the system is transferred over BLE to a PDA (smartphone or iPad), which displayed the data in a text format. This technology has some success in lowering the power consumption of an external power unit and the implanted unit. Similarly, a wireless body area network-based blood glucose level monitoring system was constructed utilizing a glucometer, an Arduino Uno, and a Zigbee module and a website was used to achieve remote glucose monitoring^25^. However, due to the high-power consumption of the Arduino Uno board and the Zigbee module, the system is not energy efficient. To address this limitation, others have employed an external transmitter to connect with and charge the glucose sensor wirelessly with Bluetooth functionality and smartphone application^26^. In addition, NFC-based devices have been proposed to improve body comfort due to its advantages of being battery-free and wireless^27, 28^. Various NFC-based applications have been demonstrated, including colorimetric sweat sensing^26^, "skin-like “device monitoring heart rate variability (HRV)^27^, epidermal ultra-violet dosimeter^28^, pulse oximetry^29^, smart-contact lenses^30^, and wireless electronic tattoo^18^.

In this work, we present a printed flexible battery-less wireless glucose monitoring system comprising a glucose abiotic hybrid fuel cell and battery-less wireless module using NFC technology. The developed anodic and cathodic electrodes are decorated with co–Pt and Ag_2_O-MWCNT composite, respectively. The voltage generated from the developed abiotic hybrid fuel cell is correlated with glucose and served as the analog signal for the data transfer from NFC module to a handheld smartphone application. The data transfer module used a low power microcontroller (MSP 430) that is enabled using a smartphone with NFC antenna receiving the respective instantaneous voltage data from the abiotic hybrid fuel cell. A developed smartphone application is deployed to communicate the data between the NFC device and the smartphone for end-user visualization and the needed energy for data transmission is provided by the smartphone. The fabricated system has the potential to be used as a tool to enable patient to record and monitor glucose levels with proper timestamp and other variables to better manage disease. In addition, the smartphone application can improve the convenience of wearable healthcare by providing personalized health information via mobile devices (e.g., smartphones and tablet PCs).

## Results and discussion

### Glucose monitoring system

**Figure 1:**
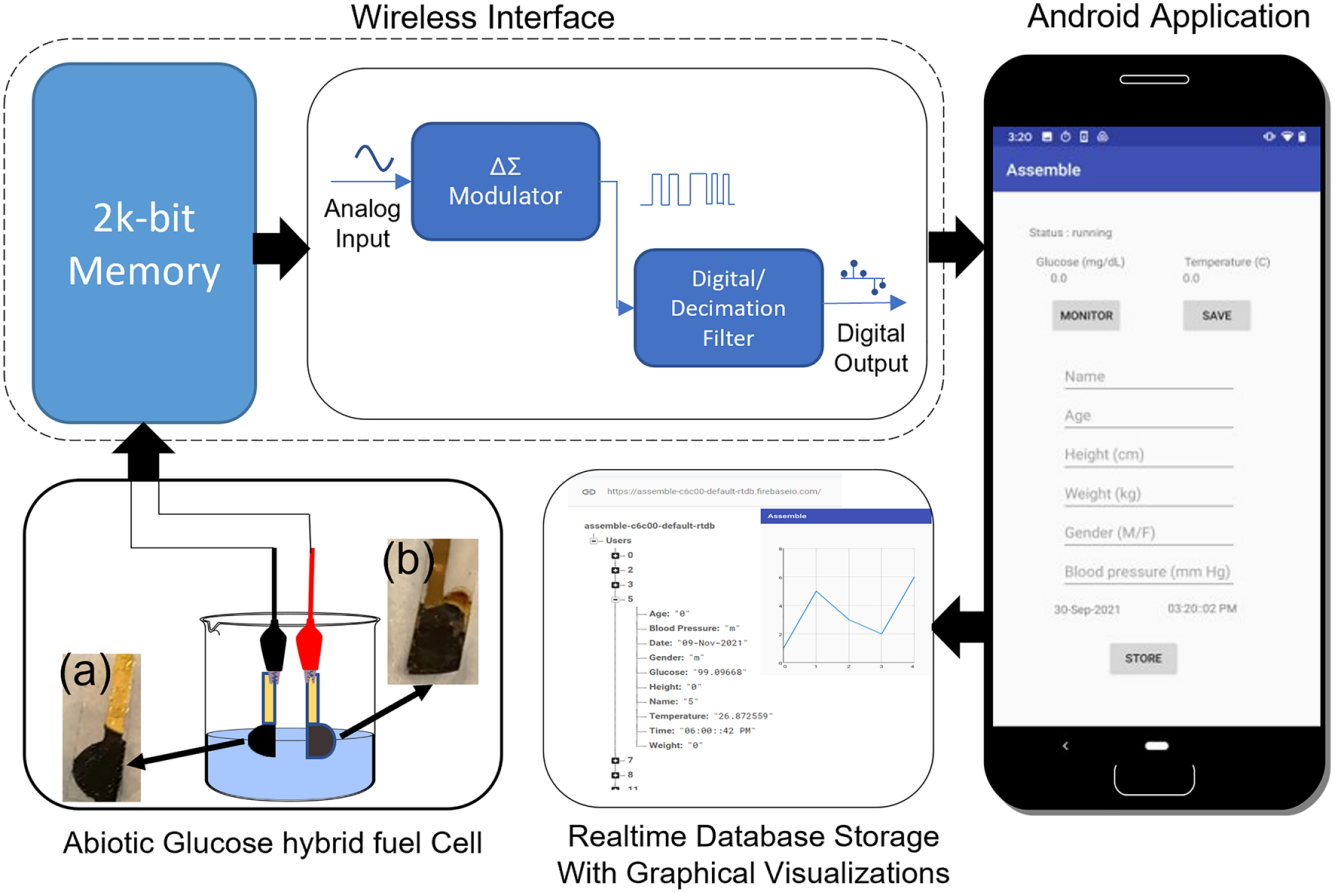
A schematic illustration of the wireless glucose monitoring system comprising an abiotic glucose hybrid fuel cell, an NFC module, and a smartphone application. The abiotic glucose hybrid fuel cell is constructed using colloidal platinum (co–Pt) anode (**a**) and silver oxide (Ag_2_O) nanoparticles-carbon nanotubes (MWCNTs) cathode (**b**).

Figure 1 schematically illustrates the glucose monitoring system based on the abiotic fuel cell, NFC module, and smartphone application. The electrode substrate material was printed on bacterial nanocellulose on polyethylene terephthalate wafer using NGP-J gold ink and a Fujifilm Dimatix 2850 Materials Printer equipped with a DMC-11610 cartridge (10 pL drop-size) following previously reported method^18^. Briefly, the printed Au anode and cathode were modified with colloidal platinum (co–Pt) and silver oxide nanoparticles-multiwalled carbon nanotubes (Ag_2_O-MWCNTs), respectively and coated with Nafion. The electrocatalytic co–Pt anode paired with the sacrificial silver oxide cathode plays a significant role in the use of the hybrid fuel cell as a glucose monitoring device since the electrical voltage correlated with the glucose concentration, and thus functions as a glucose sensor^16, 31^. The oxidation–reduction (redox) reaction of the abiotic hybrid fuel cell are as follows:

1 $$ {\text{Anode}}:Glucose + Au - co - Pt \to Gluconic \, Acid + 2H^{ + } + 2e^{ - } $$

2 $$ {\text{Cathode}}:Ag_{2} O + H_{2} O + 2e^{ - } \to 2Ag + 2OH^{ - } $$

wherein the Ag_2_O can then be slowly regenerated through exposure to air/ oxygen at room temperature^18^. The assembled abiotic glucose fuel cell was connected to the NFC (TI RF430FRL152H) module for wireless transmission.

**Figure 2:**
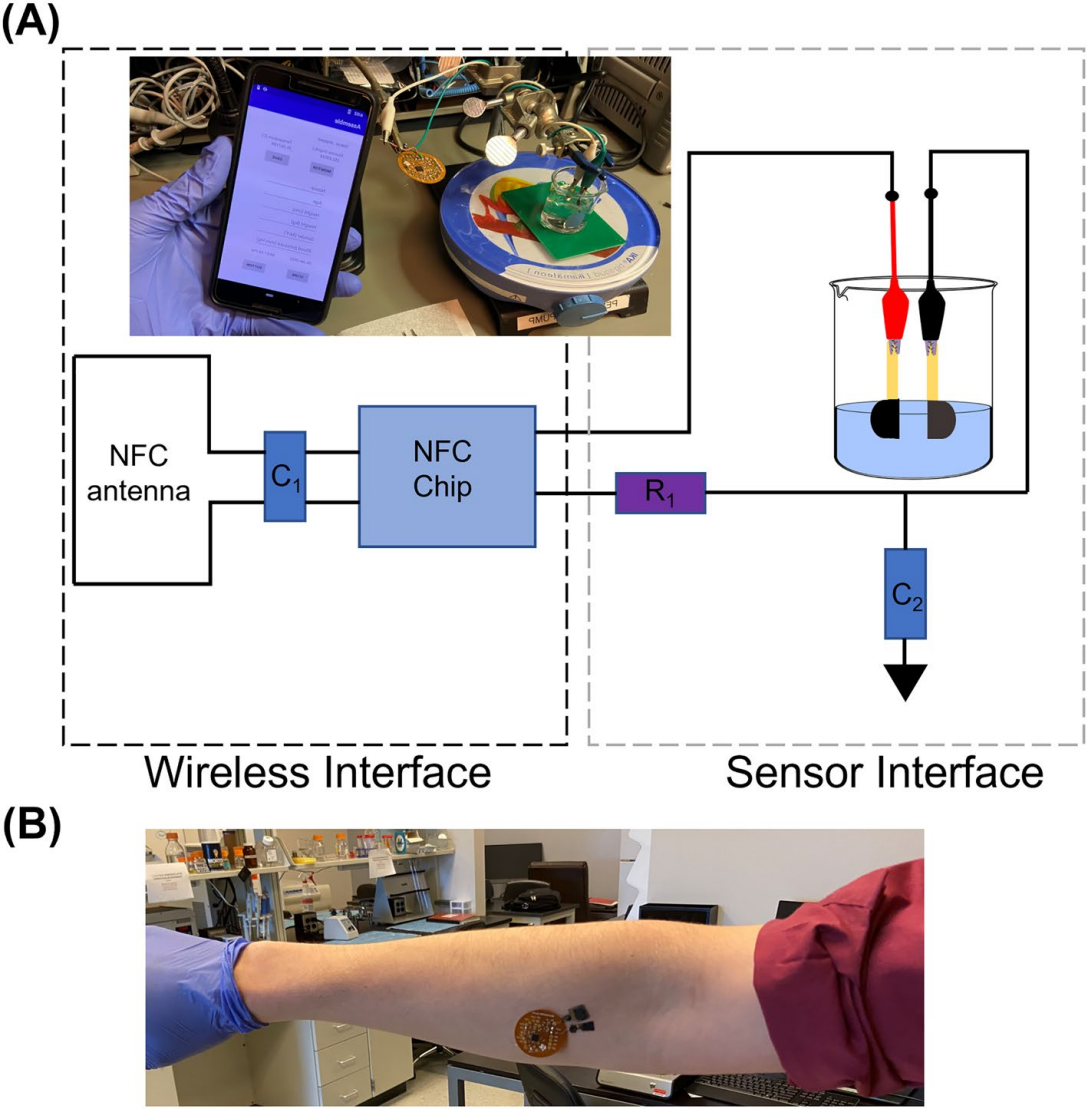
(**A**) Schematics of the glucose monitoring system comprising a glucose hybrid fuel cell and NFC based wireless module. Picture insert: Experimental setup. (**B**) The physical detection system applied to the skin.

As shown in Fig. 2A, the device operation is divided into two parts: (1) the wireless interface comprising the NFC chip and coil for wireless communication with the smartphone and (2) the abiotic glucose hybrid fuel cell interface with the NFC chip that reads the analog signal into 14-bit sigma-delta ADC values. An NFC reader, such as a smartphone is brought into proximity to the NFC module to power the device and obtain the transferred data from the NFC module. Here a resistor (R1 = 100 kΩ) was coupled to connect the fuel cell system and served as the reference resistor. Capacitors C1 (9 pF) and C2 (0.1 μF) served as the resonance capacitor for resonance frequency tuning of NFC system and the decoupling capacitor for noise removal, respectively. With the preceding analog-to-digital signal conversion in the low power microcontroller, the acquired data are transferred to the smartphone via RF field communication and converted using a custom algorithm based on the glucose calibration data. A user interface is developed by incorporating other features like age, height, weight, etc. to enable future tracking of abrupt changes that might lead to a severity in disease. The algorithm was developed in JAVA using multiple scripts to enable the extraction of the additional features for display onto a smartphone. The current android application focused on the acquisition of real-time data, providing time and date stamps, and a graphical view option for data visualization. Figure 2B shows the physical detection system applied to the skin for wearable applications.

### Electrochemical characterization

**Figure 3:**
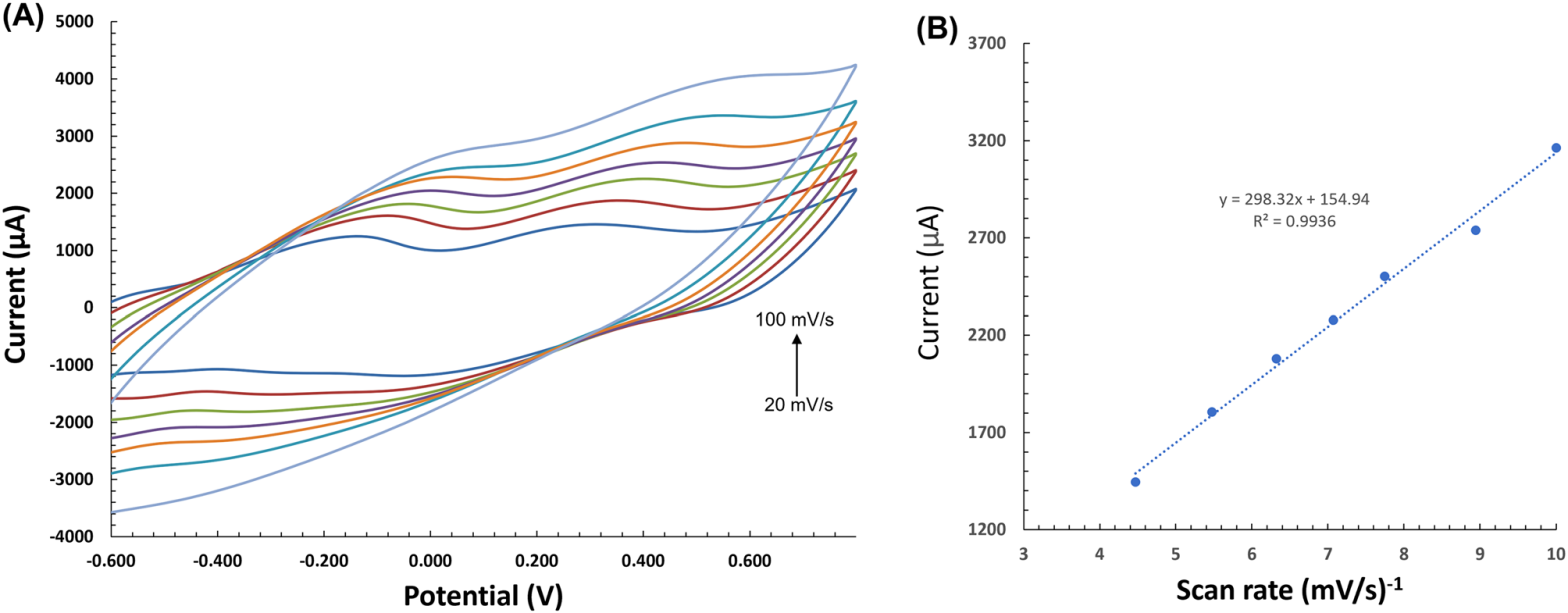
(**A**) Characterization of co–Pt Au anode by cyclic voltammetry at various scan rates in 0.1 M PBS containing 7 mM glucose. (**B**) Corresponding linear relationship between the anodic peak currents and square root of the scan rate.

To quantify the response to glucose with the constructed abiotic glucose hybrid fuel cell, cyclic voltammetry (CV) was performed for the co–Pt anode with a surface area of 1.178 cm^2^ in 0.1 M phosphate buffer solution (PBS) containing 7 mM glucose under various scan rates ranging from 20 to 100 mV/s to determine whether the electrochemical reaction is dominated by diffusion control or surface control (Fig. 3A). A potential window of − 0.7 to 0.8 V was used. Figure 3B shows that the corresponding oxidation peak current increased with increasing scan rate and is found to be linearly correlated with the square root of the scan rate and is thus diffusion controlled. The nanostructured co–Pt Au anode exhibited an effective surface area, and the observed diffusion-controlled reaction can be confirmed by the Randles–Sevcik equation, where in electrons are readily transferred between the electrolyte solution and the electrode surface^32^. The electrooxidation of the glucose is enabled by the electron transfer occurring at the gold co–Pt surface in the presence of glucose. The resultant oxidation peak exhibited higher current density around 1.435 mA/cm^2^ in comparison to the current density of 0.85 mA/cm^2^ in the absence of glucose. This shows that co–Pt exhibits a catalytic effect in the direct oxidation of glucose. The Ag_2_O-MWCNT composite is used as the electron acceptor in the hybrid fuel cell as depicted in Eq. (2). Ag_2_O is reduced to Ag in air at an onset potential of 0.231 V. In addition, this sacrificial Ag_2_O cathode can then be slowly regenerated through exposure to air/ oxygen in PBS at room temperature. Linear sweep voltammetry is used to obtain the performance characteristics of the abiotic hybrid fuel cell. An open circuit voltage of 0.46 V, short circuit current density of 0.444 mA/cm^2^, and maximum power density of 0.062 mW/cm^2^ at 0.26 V in the presence of 7 mM physiologic glucose are obtained, thereby indicating successful electron transfer for the co–Pt and Ag_2_O-MWCNTs electrodes.

**Figure 4:**
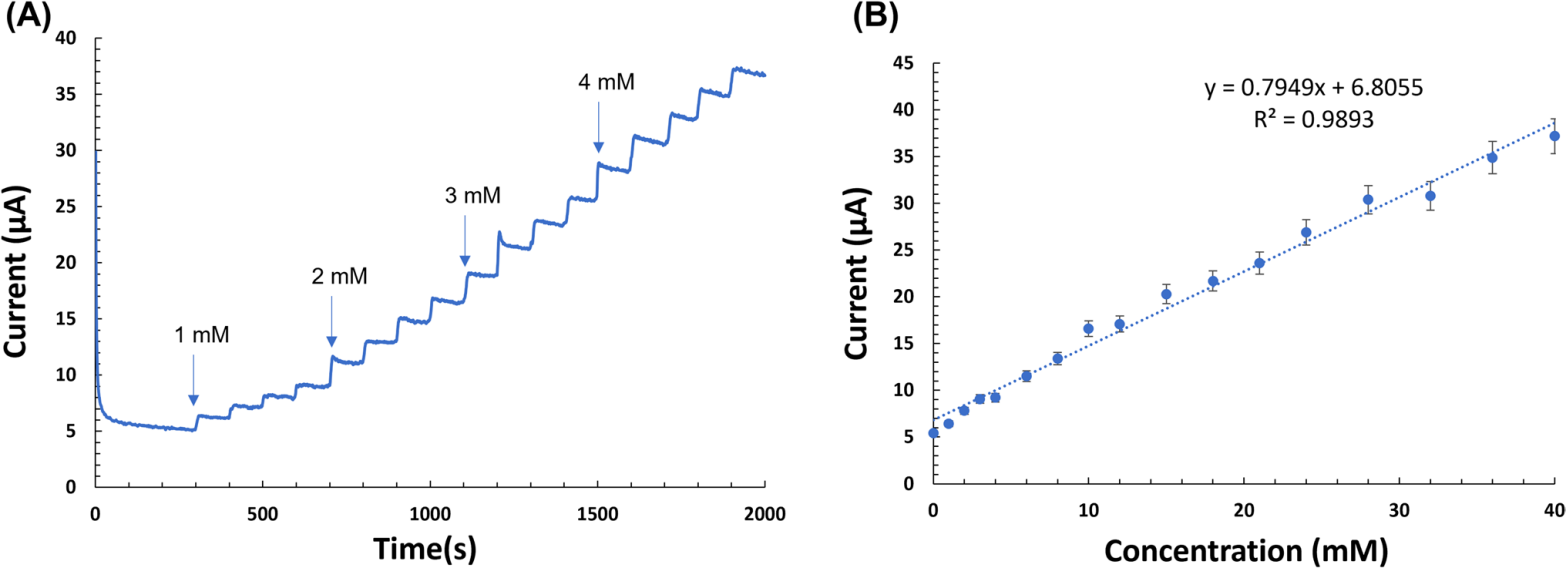
(**A**) Chronoamperometry of co–Pt upon successive additions of glucose (applied potential: − 0.1 V) in 0.1 M PBS pH 7.4. (**B**) Corresponding calibration curve. Error bars: ± standard deviation of triplicate measurements.

**Figure 5:**
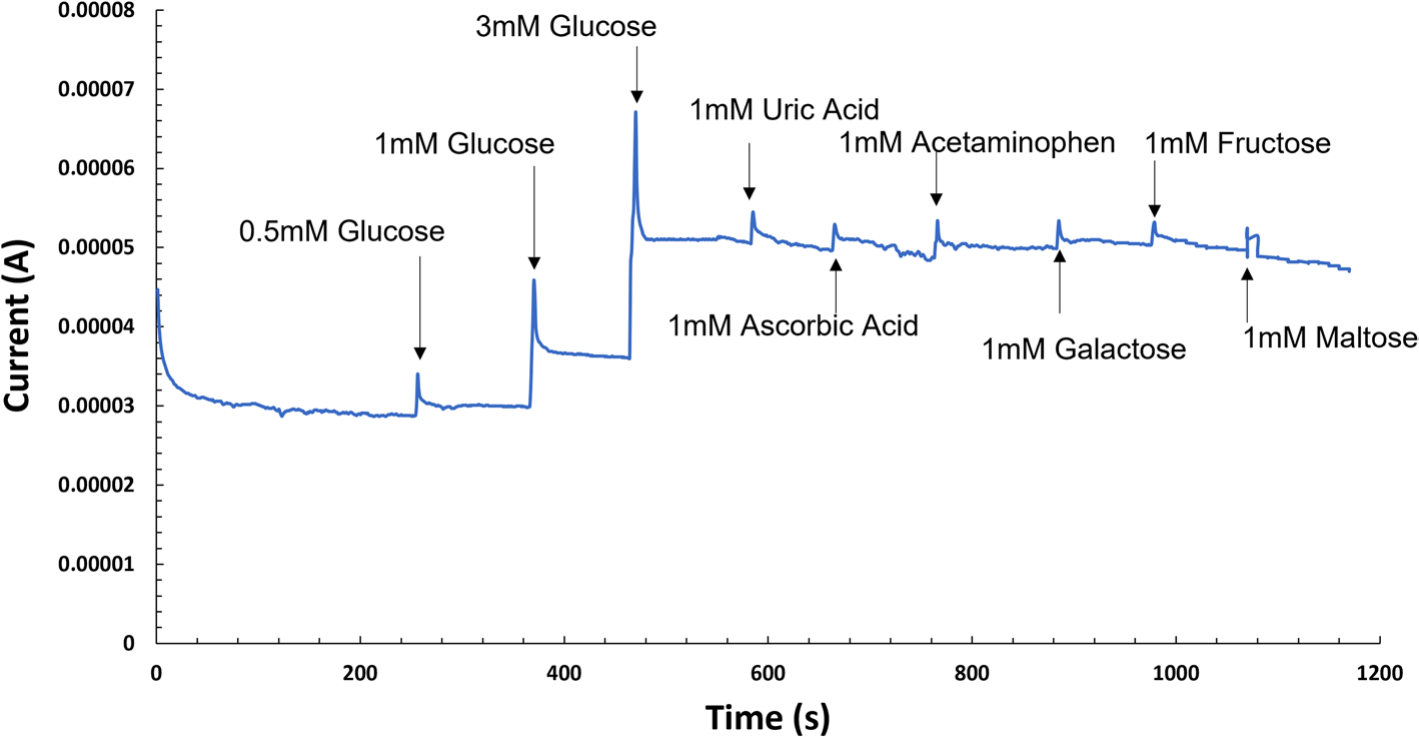
Current (*i*) response profile of interfering analytes at co–Pt anode upon the addition of 0.5 mM, 1 mM, and 3 mM glucose and 1 mM of interfering analytes at applied potential: − 0.1 V in 0.1 M PBS pH 7.4.

Chronoamperometry of the co–Pt Au anode was performed in 0.1 M phosphate buffer supporting electrolyte under constant stirring and followed by consecutive addition of glucose at an applied potential of − 100 mV. Figure 4A shows the stepwise staircase amperograms upon successive addition of glucose. Upon the addition of the glucose aliquot, the oxidation current increased nearly instantaneously to a steady-state oxidation current within 3 s. The rapid sensor response is due to catalytic activity of the co–Pt, in which electrons are immediately transported to the electrode surface from the glucose electrolyte^33^. The oxidation currents increased with increasing glucose concentration. Figure 4B shows that the corresponding calibration curve exhibits a linear relationship up to 40 mM glucose (r^2^ = 0.989) with a sensitivity of 0.795 mA/mM•cm^2^. Competing and non-competing analytes co-exist with glucose and are easily oxidized. The interfering analytes in biological fluids are at least 10 times lower than that of glucose. Figure 5 shows the current response curve upon the addition of glucose (0.5 mM, 1 mM, and 3 mM) and 1 mM of competing and noncompeting interfering analytes (uric acid, ascorbic acid, acetaminophen, galactose, fructose, and maltose) at the impressed potential of − 0.1 V. The response current increased sharply and reached a steady state for glucose. The current responses for uric acid, ascorbic acid, acetaminophen, galactose, fructose, and maltose were observed to be insignificant^5^. These results indicate good selectivity of the co–Pt, which is attributed to the nafion coating and specificity enabled by the co–Pt.

### Battery-less wireless glucose monitoring system

The TI RF430FRL152H uses an NFC/RFID communication to transmit data wirelessly over short distances typically ranging from 1 to 5 cm^34^. The NFC is a passive 13.56 MHz RFID transponder chip that contains an ISO 15,693 and ISO 180,003 compliant RFID interface as well as a programmable 16-bit microcontroller MSP430 with 2 KB integrated Ferroelectric Random-Access Memory (FRAM), and sigma-delta analog-to-digital converter (ADC) interfaces. The NFC module was implemented as it explores the electromagnetic induction between two coil-type inductors, where the induced power is maximized by matching the resonance frequency of the two coils in the NFC device and reader (e.g., smartphone), respectively^35, 36^. Higher inductance is necessary for stronger magnetic power induction, which is mostly dictated by the number of turns^37^. Because a higher number of turns necessitates a bigger dimension, device miniaturization and high magnetic power are mutually exclusive. The antenna used has 5 turns with 0.5 mm spacing between adjacent turns. Moreover, the NFC reader not only serves as a power source for the NFC device, but it can also read data from it^38, 39^. In this case, an NFC-enabled smartphone can power the NFC device^40, 41^. The electrodes of the abiotic glucose hybrid fuel cell connected to the NFC module were immersed in an air-saturated glucose solution. The power transmission began the measurement and the time taken to receive the transmitted data was less than 2 s. The voltage generated by the abiotic glucose hybrid fuel cell is transmitted through the NFC module, which then converts the voltage analog signal to digital signal for low voltage signals from 0.0 to 0.9 V. The built-in sigma ADC conversion of the collected signal is then transmitted to a smartphone through radiofrequency (RF) field communication to enable the developed glucose correlation algorithm to calculate and report the detected glucose value. This data is further processed to provide real-time reporting. Thus, the NFC enabled the wireless measurements of the electrochemical abiotic glucose hybrid fuel cell.

The NFC device has an integrated, universal, non-volatile FRAM memory for storing program code or user data such as calibration and measurement data. The built-in 14-bit sigma-delta ADC has an analog front end that includes a with programmable gain amplifier so that the input signal does not reach the upper limits of the input power. The sigma-delta modulator scans the analog input signal and reduce the noise at lower frequencies. In this case, the built-in ADC enabled high resolution analog to digital conversion of the captured sensor readings with sampling frequency of up to 2 kHz. We used a Google Pixel 3a smartphone as the NFC reader due to its compatibility with ISO/IEC 15693 module to supports contactless communication over the analog front end^35^. The Google Pixel 3a with ISO/IEC 15693 capability was configured for the device to run and when the device completes the configured number of sensor scans, it turns itself off. The NFC device can be restarted by applying an RF field again.

**Figure 6:**
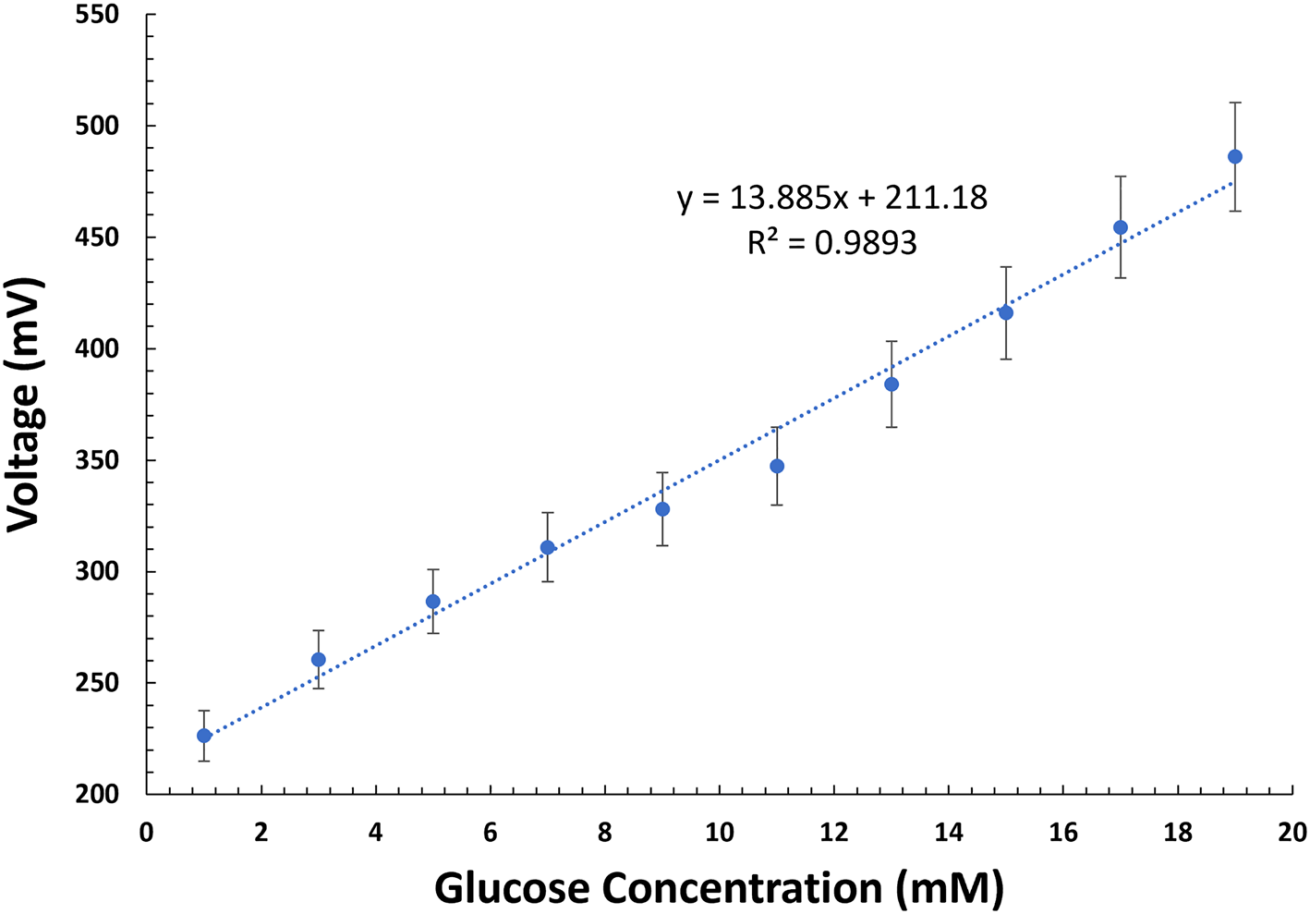
Glucose hybrid fuel cell voltage-glucose linear relationship.

The voltage supplied to the NFC circuit by the abiotic glucose hybrid fuel cell is recorded and transmitted wirelessly. The data received is stored in raw format which is fed through the modulator outputs namely at high frequency and 1-bit output speed^38^. A low pass digital filter function is used to attenuate high frequency noise resulting in a high-resolution signal from the abiotic glucose hybrid fuel cell. The processing was achieved using an android studio-based development tool, in which a custom algorithm was developed to take the saved data and categorize them into the three ADC inputs that are available on the MSP430 microcontroller. The microcontroller is coded to read the fuel cell analog voltage signals in response to consecutive addition of glucose in mg/dL. The best fit regression line graph (Fig. 6) shows a linear correlation between the fuel cell voltage value and the glucose level (1–19 mM) with correlation coefficient, r^2^ = 9893. This linear correlation enables the glucose level to be computed via the linear equation: y = 13.885x + 211.18. Supplementary Figure S1 shows an example of the script used to convert the digital values to the respective glucose concentrations. The recorded data is stored in the firebase real-time database along with the time stamp and user input parameters. The firebase database is a NoSQL based cloud storage that allows access to the data across different platforms. The database can be used offline and to capture the data in device memory and synchronizing it after reconnecting to the internet. Parameter such as age, height, weight, and time stamp are important parameters that can be eventually used to provide better diabetes management in terms of the risk of reaching the upper or lower limits of glycemic ranges.

**Figure 7:**
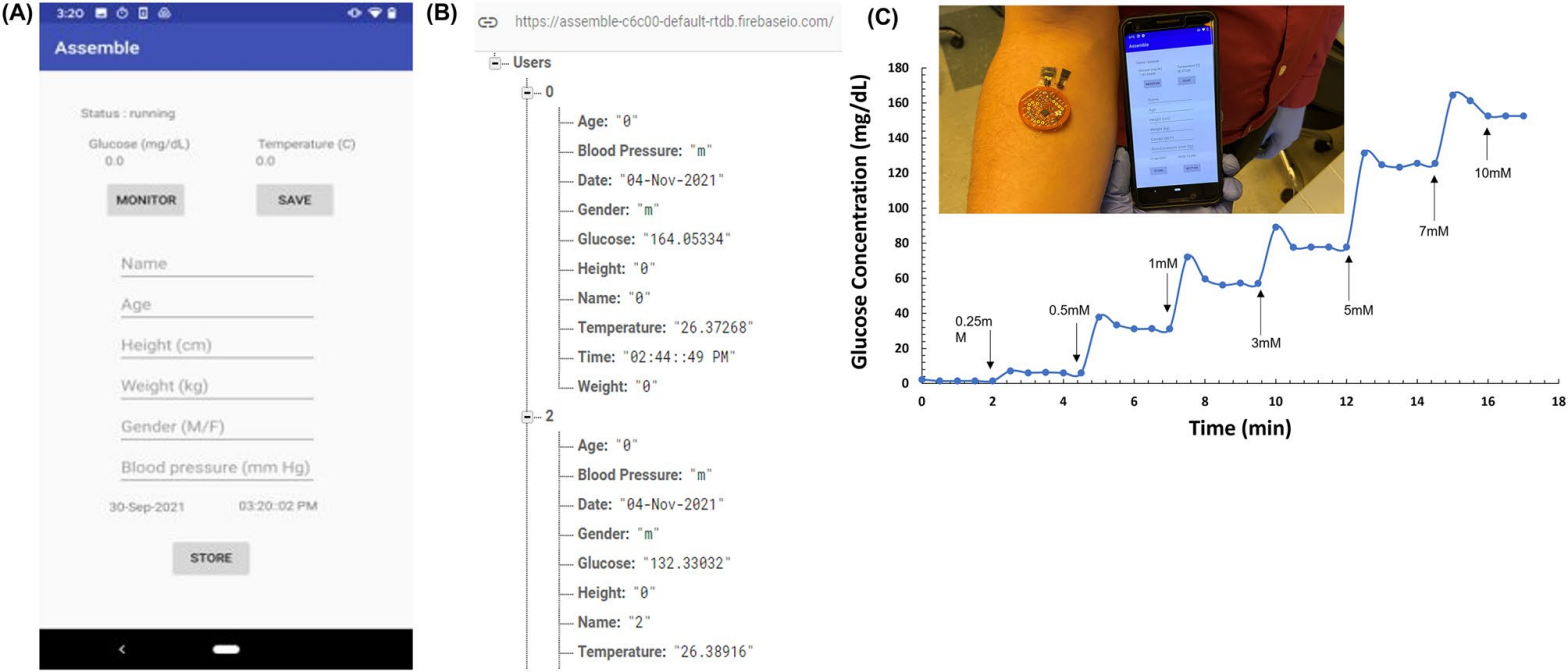
(**A**) Smartphone interface for glucose and other independent feature capture (**B**) Firebase Realtime cloud database. (**C**) Stability profile of glucose monitoring system operating on increasing concentration of glucose [0.25–10 mM] in 0.1 M PBS pH 7.4. Insert picture: Physical detection system exposed to standard glucose solution (7 mM).

The measurements taken by the microcontroller is sent to the smartphone for visualization. The application (.apk) with the name of “Assemble” is created and the main GUI of the created application is shown in Fig. 7A with the user input parameters and glucose value obtained from glucose monitoring system. Figure 7B provides a screenshot of the data storage in the firebase cloud database. Each user is assigned an autogenerated user ID and the real time measurement is recorded to the database stored under the specific user ID. The glucose hybrid fuel cell connected to the NFC circuit maintains an output voltage between 0.22 and 0.7 V during operation. We recorded the glucose concentration output of the glucose monitoring system as a function of time upon successive addition of glucose using the smartphone application and Fig. 7C shows the stability in the response of the system in 0.1 M PBS over 17 min. The response of the glucose monitoring system upon successive addition of varying glucose concentrations (0.25–10 mM) remained stable until the next aliquot was added after the 2 min period, after which its performance immediately shot up followed by a gradual decline after ~ 30 s to reach a steady-state level. The insert picture provides a proof-of-concept glucose detection. This result confirms that the wireless glucose monitoring system is promising and desirable for glucose monitoring applications.

In the management of glucose, variability in the overall fasting glucose levels and glucose levels in diabetes individual when compared with normoglycemic or prediabetic individuals have been observed^39^. The terms ‘hyperglycemia’ and ‘hypoglycemia’ are traditionally used to describe glucose levels that are ‘too high’ or ‘too low’, respectively, in Type 1 and Type 2 diabetes management^40, 41^. This scenario is shown in Supplementary Figure S2, where the glucose monitoring system is exposed to various glucose concentrations generated using a random number generator to mimic glycemic responses. Some of the responses were sufficiently high and low as to be characterized as hyperglycemia or hypoglycemia. For self-management of glucose, the red lines provide the lower and higher threshold boundary in which to maintain glucose fluctuations and persistent fluctuations above or below these thresholds over time may result in hospitalization^42^. These results demonstrate that the battery-less wireless glucose monitoring system can detect transient changes in glucose level and tracking of weight and stress-related parameters that affect blood glucose. The developed glucose monitoring system has the potential to enable user accountability in the self-management of glucose and to make necessary lifestyle changes to improve care.

In summary, we developed a proof-of-concept flexible battery-less wireless glucose monitoring system comprising an abiotic glucose hybrid fuel cell, an NFC module, and a smartphone application. The application framework included a personalized glucose tracking. The glucose hybrid fuel cell generated an open circuit voltage of 0.46 V, short circuit current density of 0.444 mA/cm^2^, and maximum power density of 0.062 mW/cm^2^ at 0.26 V in the presence of 7 mM physiologic glucose. A linear range of 1–19 mM glucose with a sensitivity of 13.9 mV/mM•cm^2^ was observed for the glucose monitoring system. The inclusion of the NFC wireless module with ADC conversion capability enabled the detection of the fuel cell voltage in response to glucose to be converted to a digital output for a smartphone application. The smartphone application was designed to record the data generated from the glucose monitoring system and provide real-time visualization of the measured data. We demonstrated the successful operation of the glucose monitoring system to output stable glucose readings and to respond to transient changes in glucose level. Furthermore, a systematic screening for high glycemic fluctuation would allow for the early identification of individuals at risk of avoidable diabetes complications. Preventing diabetes complications increases patients' quality of life while lowering the financial burden of health-care expenses. Future work will include the creation of a predictive software package for clinical usage as well as the exploration of the system in biological fluids.

## Methods

### Materials

Silver nitrate, polyethylene glycol 3000 (PEG), sodium hydroxide, D ( +) glucose, potassium phosphate monobasic, sodium azide, uric acid, ascorbic acid, acetaminophen, galactose, fructose, maltose, and Nafion were obtained from Sigma-Aldrich. The platinizing solution was purchased from YSI Inc., and NGP-J gold nanoparticle ink was acquired from Iwatani Corporation of America. The multiwalled carbon nanotube NINK-1000 was obtained from Nanolab, Inc. The bacterial nanocellulose (BNC) was synthesized using previously reported method using Gluconacetobacter xylinus (ATCC 10245) culture in hydrosulphite of sodium medium (HS medium)^18^. All the solutions were prepared with 18.2 MΩ-cm Milli-Q water. Platinum counter electrode, Ag/AgCl reference electrode, and PalmSense4 potentiostat were purchased from BASI Inc. Texas Instruments (TI) RFID transponder integrated circuit (IC) RF430FRL152H was purchased from the Digi Key Electronics and a Google Pixel 3A served as the smartphone device.

### Electrochemical measurements

All electrochemical measurements (cyclic voltammetry and chronoamperometry) were conducted using Palmsense4 electrochemical workstation. The electrochemical cell consists of a conventional three electrode system where the gold printed modified electrode was used as the working electrode, Ag/AgCl (3 M KCl) electrode as the reference electrode, and a platinum electrode as the counter electrode for characterizing the electrode in 0.1 M potassium phosphate buffer (pH 7.4) containing various glucose concentrations. All electrochemical experiments were carried out at room temperature (25.0 ± 0.5 °C) using an electrochemical cell.

### Glucose monitoring system

Briefly, the printed Au anode was modified via electrodeposition of colloidal platinum (co–Pt) using YSI platinizing solution at an applied potential of − 225 mV vs. Ag/AgCl for 1500 s. The printed Au cathode was modified with silver oxide nanoparticles-multiwalled carbon nanotubes (Ag_2_O-MWCNTs). The Ag_2_O nanoparticles were synthesized from a solution of PEG and silver nitrate at 75 °C and pH 9.8 for 1 h. A solution of MWCNTs was mixed in with the Ag_2_O nanoparticles via ultra-sonication to realize the Ag_2_O-MWCNTs deposited on the printed gold surface. The nanostructured printed Au anode and cathode were coated with Nafion to separate the anode and cathode and was employed to enhance the stability of the nanocomposite as well as to selectively screen against interfering analytes. The constructed electrodes are assembled by bonding the bond pads via carbon glue to a tungsten wire, which was then sealed with wire glue. The catalytic efficiency of the abiotic glucose hybrid fuel cell was enhanced using the nanostructured anode and cathode. The assembled abiotic glucose fuel cell was connected to the NFC (TI RF430FRL152H) module for wireless transmission. Figure 2 picture insert shows the overall experimental setup. The on-body study was performed under a protocol approved by the institutional review board (IRB) at Old Dominion University (IRB19065965). All experiments were performed in accordance with guidelines and regulations. All participants read the study description document and provided written informed consent before participation. Additionally, participants completed a health screening questionnaire to ensure the absence of any heart or lung conditions, or other conditions that may alter their ability from exercising and breaking a sweat.

## Supplementary Information

Supplementary Information.

## Data Availability

The datasets used and/or analyzed during the current study available from the corresponding author on reasonable request.
