# Supplementary material for: Flexible battery-less wireless glucose monitoring system
Source: Sci Rep. 2022 Jul 19;12:12356. doi: 10.1038/s41598-022-16714-1 (PMC9296503; doi:10.1038/s41598-022-16714-1)
Supplement: Supplementary file 1 — Supplementary Information. [file 41598_2022_16714_MOESM1_ESM.docx]

Flexible Battery-less Wireless Glucose Monitoring System

*Saikat Banerjee and Gymama Slaughter**

Center for Bioelectronics, Department of Electrical and Computer Engineering, Old Dominion University, Norfolk, VA-23528, USA

**Supplementary Information**


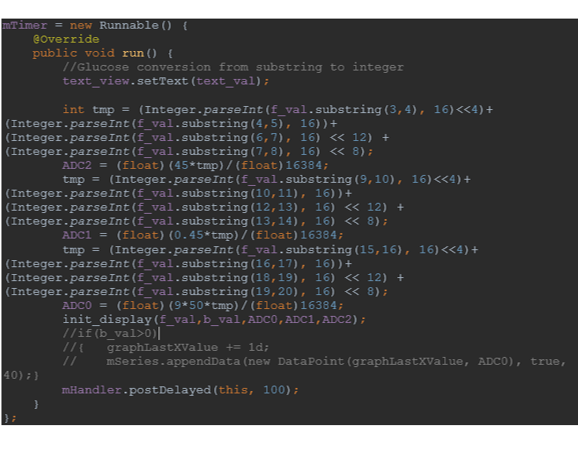


**Fig.S1.** Screenshot of the script used to convert the digital values to the respective glucose concentrations.


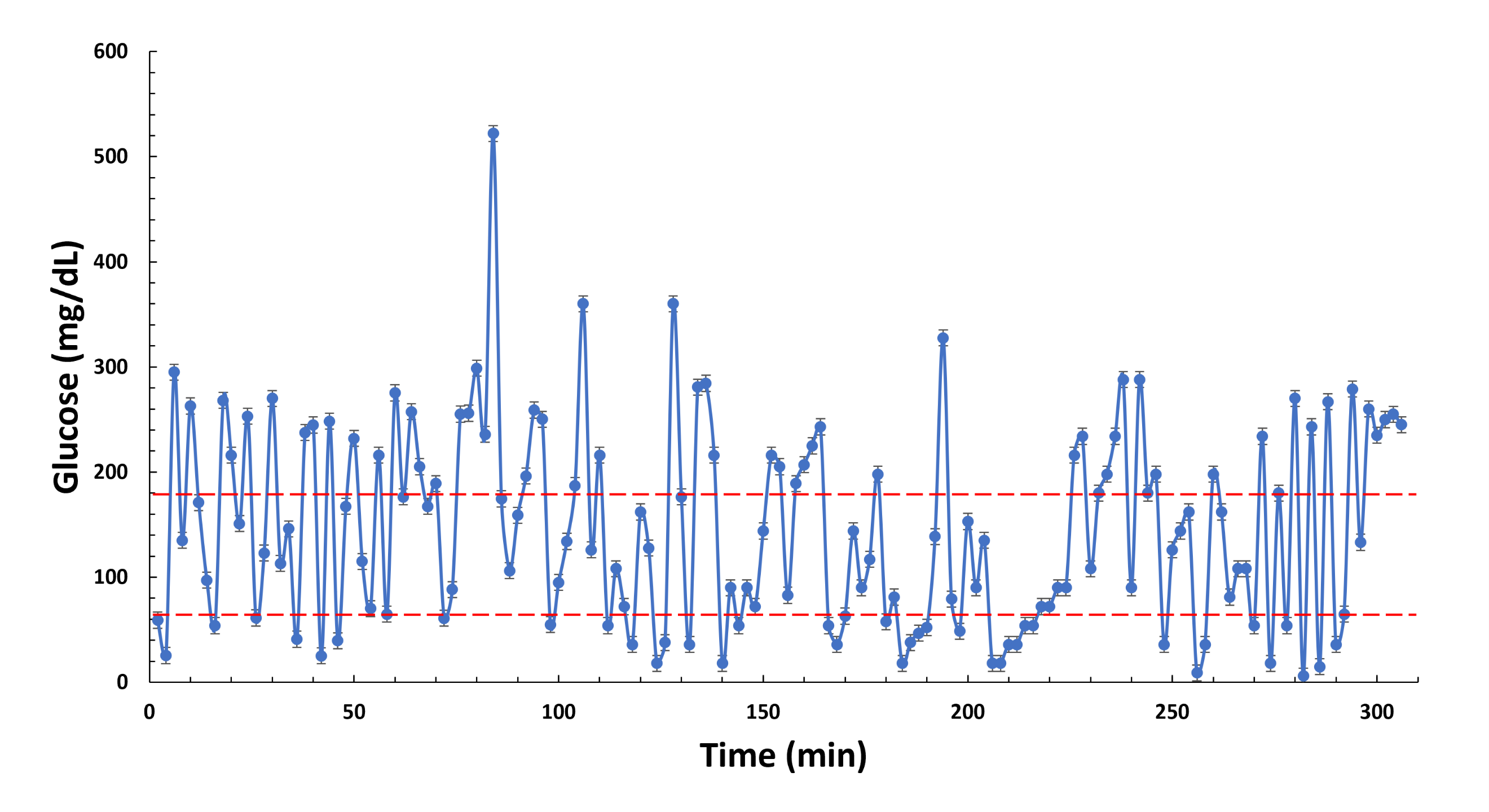


**Fig. S2.** Mimicking glycemic responses by exposing the glucose monitoring system to various glucose concentrations generated using a random number generator. Respective glucose aliquot was added after 3-min interval. Error bars represent ± standard deviation of triplicates. measurements.
